# Bioactive Polymeric Nanoparticles of *Moringa oleifera* Induced Phyto-Photothermal Sensitization for the Enhanced Therapy of Retinoblastoma

**DOI:** 10.3390/pharmaceutics15020475

**Published:** 2023-01-31

**Authors:** Sushma Venkata Mudigunda, Deepak B. Pemmaraju, Sri Amruthaa Sankaranarayanan, Aravind Kumar Rengan

**Affiliations:** 1 Department of Biomedical Sciences, Indian Institute of Technology Hyderabad, Kandi 502284, India; 2 Department of Pharmacology & Toxicology, National Institute of Pharmaceutical Education & Research (NIPER), Guwahati 781101, India

**Keywords:** *Moringa oleifera*, phyto-photothermal therapy, retinoblastoma, polymeric nano system, heat shock proteins

## Abstract

Treatment of retinoblastoma is limited due to its delayed detection and inaccesbility of drugs to reach the retina crossing the blood-retinal barrier. With the advancements in nanotechnology, photothermal therapy (PTT) employing plasmonic nanomaterials and/or NIR dyes have emerged as an affordable alternative owing to the spatial control that is offered by the modality leading to localized and enhanced therapeutic efficacy with minimal invasiveness. However, the modality is limited in its clinical application owing to the increased heat shock resistance of the tumor cells in response to the heat that is generated via PTT. Hence, in this study, we explore the role of novel biomolecular fraction of *Moringa oleifera* (DFM) encapsulated within a polymeric nanosystem, for its anti-heat shock protein (HSP) activity. The MO extract was co-encapsulated with NIR sensitizing dye, IR820 into a biodegradable polycaprolactone (PCL) nano-delivery system (PMIR NPs). The photothermal transduction efficacy of PMIR NPs was validated in vitro against retinoblastoma cell lines. The inherent fluorescence of DFM was utilized to evaluate the cellular internalization of the PMIR NPs using fluorescence microscopy and flow cytometry. The overall oxidative protein damage and downregulation of HSP70 expression upon treatment with PMIR NPs and NIR laser irradiation was evaluated using densiometric protein analysis and Western blotting. Overall, the PMIR NPs exhibited excellent anti-cancer activity when combined with PTT with downregulated HSP70 expression against retinoblastoma cells.

## 1. Introduction

Cancer is one of the major contributors of the world’s mortality rate. A total of 4.4 million cases of out 9.9 million cases reported globally end up in death owing to the aggressive nature of cancer [1]. Retinoblastoma is a form of intraocular malignancy, most commonly affecting children, representing 4% of all childhood cancers. The cause of origin is mainly due to loss of function or mutation of the Rb tumor suppressor gene [2,3,4]. The conventional chemotherapy is majorly challenged by the blood-retinal barrier which limits the accessibility of drugs to the eyeball. Development in localized delivery of drugs via intraarterial chemotherapy or via direct intravitreal delivery of chemotherapy drugs also are limited by the requirement of specific equipment and great expertise from the medical team as these methods have all the possibilities of fatal side effects such as hemorrhage, endophthalmitis, and retinal detachment. The effectiveness of the treatments is significantly limited due to the delicate anatomical structure of the site and potential side effects [5,6,7,8]. Another critical challenge in the treatment of retinoblastoma is the severe cytotoxicity of the drugs that are conventionally used such as melphalan, topotecan, and carboplatin [9,10,11,12]. Focal therapy, employing a diode laser focusing directly on the tumor, is being used to eliminate tumor cells specifically. However, these therapies demand extreme caution as they are highly prone to damaging the optic disc and neurosensory papillomacular bundle, in turn leading to secondary malignancies [13,14]. Hence, development of simple, yet affordable treatment modalities with increased therapeutic efficacy and minimal/no adverse side effects is warranted.

Photothermal therapy (PTT), in this perspective, has emerged as an affordable alternative to the existing treatment strategies owing to its minimally invasive nature with high efficiency and specificity in tumor cell destruction [15]. The spatial control, in addition to its penetration depth, offered by PTT is utilized to a large extent in the treatment of superficial tumors to avoid damage to the surrounding healthy tissues [16,17]. PTT employs plasmonic metallic nanosystems or NIR dye, which convert the incident NIR light into light, thereby causing a localized ablation of the tumor cells [18,19,20]. Several studies have reported the use of photothermal therapy for the treatment of retinoblastoma [21,22,23,24,25,26,27,28]. However, recent literature reports reveal that the tumor cells acquire inherent resistance to the heat that is induced via PTT through the activation of heat shock proteins (HSP). This leads to thermo-resistance leading to compromised therapy in the majority of the cancers, thereby limiting its clinical applications [29,30,31,32]. 

Despite significant improvement in the field of drug discovery and development for cancer treatment, a major concern always arises regarding the adverse side effects and toxicity that is caused by these chemo drugs or radiation-based effects. The most used chemotherapeutic drugs such as 5-Fluorouracil, Doxorubicin, and Cyclophosphamide has been reported for their cardio, renal, bladder, and pulmonary toxicities [33,34,35,36,37]. This leads to a quest for naturally occurring substances as novel formulations against the fatal disease. Exploring the use of medicinal plants and their extracts in the treatment of various diseases dates back to centuries ago. Plant-derived drugs have also been used in market (mainly alkaloids, phyllotoxins, taxanes, and camptothecin derivatives). Several parts of a plant, such as bark, leaves, fruit body, seeds, etc. have been investigated for their bioactive fractions/components having effective anti-cancer properties [38,39]. Extracts of *Moringa oleifera* have been known to exhibit excellent medicinal properties such as antioxidant, anti-inflammatory, anti-cancer, cardiac, and hepatoprotection, etc. Recently, a study by Tekayev et al., reported the role of *M. oleifera* in modulating the expression of HSP70 in rat models of cryptorchidism [40]. Several studies have also reported the use of *M. oleifera* extracts in anti-cancer applications through downregulation of tumor suppressor genes and apoptotic markers [41,42,43,44]. However, to the best of our knowledge, the role of *M. oleifera* extract in downregulation of HSP70 expression to synergistically support photothermal therapy is underexplored.

In this regard, identification and delivery of affordable and dietary HSP inhibitory molecules that can surmount the tumor thermoresistance would be of great value in improving the overall efficacy of PTT. Herein, we have entrapped a bioactive fraction from *Moringa oleifera* that was identified to inhibit the heat shock response in the retinoblastoma cell lines and therefore combined along with the photothermal therapy to enhance the therapeutic outcome. Polycaprolactone-based nanoparticles were synthesized by entrapping the NIR dye, IR820, and the bioactive heat shock inhibitory fraction from the DFM. The NPs without encapsulation of the IR820 or DFM were also prepared as respective controls. The cellular uptake pattern and the resultant nanoparticle’s photothermal efficacy was evaluated in retinoblastoma cell line models. 

## 2. Materials and Methods

### 2.1. Materials

*Moringa oleifera* leaves were procured from the local market. A total of 500 g of leaves were thoroughly air-dried and then ground into powder using an electric grinder. Polycaprolactone (PCL; MW 10 KDa), polyvinyl alcohol (PVA, MW 10 KDa), bovine serum albumin (BSA), IR820 (NIR dye), Fluorescein diacetate, propidium iodide (PI), resazurin, DAPI, MTT (3-(4,5-Dimethylthiazol-2-yl)-2,5-Diphenyltetrazolium Bromide), bicinchoninic acid (Cat No. BCA1), were purchased from Sigma (St. Louis, MO, USA). pSuper (Plasmid #14581), GAPDH (mAb #2118) and HSP70 (mAb #4872) antibodies were purchased from Cell Signaling Technologies. Solvents such as dimethyl sulfoxide (DMSO), chloroform and dichloromethane (DCM), were procured from SRL chemicals. RPMI-1640, trypsin-EDTA, fetal bovine serum (FBS, US origin), and phosphate buffer pH 7.0 were purchased from Hi-media Chemicals, India. The retinoblastoma cell line (Y79) and HRMEV (human retinal microvascular endothelial cells) were obtained from the Centre for Cellular and Molecular Biology (CCMB), Hyderabad, India. Mouse fibroblast cell lines (L929) were purchased from National Centre for Cell Sciences (NCCS), Pune, India. 

### 2.2. Methods

**Sample Preparation:** Initially, n-Hexane solvent along with the coarse power of *M. oleifera* was heated at 80–90 °C for 5–8 h in a Soxhlet apparatus. The pure filtrate was collected in a flask with a circular bottom. During the solvent fractionation, the filtrate was dried and equal parts of dichloromethane (DCM), chloroform, and acetone were added subsequently, and the extracts were condensed by a rotary evaporator. These were labeled as HFM-n-hexane fraction, DFM-di-chloromethane fraction, CFM-Chloroform fraction, and AFM-Acetone fraction of *Moringa oleifera*. which were then kept at 4 °C for future work.

**Screening of bioactive components:** Each sample was examined to estimate alkaloids, saponins, flavonoids, tannins, and sterols using different protocols, and readings were taken in triplicate to validate the mean value [45]. Gas chromatography–mass spectrometry analysis of Dichloromethane extract of *Moringa oleifera* leaf was done with Shimadzu Japan gas chromatography QP2010PLUS with a fused GC column (2010) and coated with polymethyl silicon (0.25 nm × 50 m). The components of the extracts have been provisionally identified by corresponding peaks using Computer Wiley MS libraries, and these identifications have been validated by comparison with peaks of mass spectra that have been reported in published literature.

**DPPH assay:** Radical scavenging activity of samples was estimated using DPPH radical scavenging assay [46]. The free radical, liberating plant extract was calculated by decolorizing 2,2-diphenyl-1-picrylhydrazyl solution (65). It develops purple color in methanol. A total of 2.4 mL of 0.1 mM DPPH solution was combined with 1.6 mL of a leaf at increasing concentration (12.5–150 μg/mL). The solution was mixed and kept in the dark for 30 min. The absorbance was measured at 517 nm. 

**Characterization of Fractions of *Moringa oleifera*:** The absorbance and fluorescence spectra of DFM, fractions of *Moringa oleifera* were estimated using a UV-visible spectrophotometer (UV-1800, Shimadzu, Japan) and fluorescence spectrophotometer (RF-6000, Shimadzu, Japan). The images of fluorescence of the fractions of *Moringa oleifera* DFM were taken.

**Cytotoxicity of fractions of *Moringa oleifera*:** Cytotoxicity was studied in HRMEV (human retinal micro vascular endothelial cells) and Y79 (Retinoblastoma) using MTT assay. A total of 1 × 10^4^ cells per well of HRMEV and Y79 cells were seeded in a 96 well plate. Post 24 h, a fraction of *Moringa* (DFM) at 25–1000 μg/mL concentration was added to the media. After 48 h of incubation, cell viability was calculated using an MTT assay. 

**Synthesis and characterization of nanoparticles:** Nanoparticles were synthesized using polycaprolactone (PCL) polymer by the process of emulsification diffusion along with solvent evaporation technique [47]. PCL NPs are loaded individually with DFM (M PNPs), IR-820 (I PNPs) and DFM/IR-820 (MI PNPs). A total of 5 mg of PCL was mixed with dichloromethane and added to 1% *w*/*v* PVA solution (10 mL) (Daihan homogenizer, Korea). The sample was further sonicated for 3 min at 55 W. The samples were purified by centrifugation, followed by lyophilization, and then stored at 4 °C. M PNPs, I PNPs, and MI PNPs were synthesized using 2 mg of DFM and IR-820 mixed with 5 mg PCL. The synthesized nanoparticles were characterized for their UV-Visible absorbance (UV-1800, Shimadzu, Japan), mean hydrodynamic diameter (Beckman Coulter, Delsa Nano C), size, and morphology using TEM (JEOL, JEM 2100, USA) and FE-SEM (Carl Zeiss atomic microscope, Supra-40, Jena, Germany). The fluorescence of the synthesized nanoparticles were recorded using Enspire multimode plate reader (Perkin Elmer, USA).

**Cellular uptake studies of MI PNPs:** The cellular internalization of PCL-DFM in Y79 cells was evaluated using fluorescent microscopy. Briefly, the cells were plated at a density of 2 × 10^5^ cells/well. Post 24 h of incubation, the cells were treated with PMIR NPs for 8 h. The cells were washed with 1X PBS and fixed with formaldehyde (4%) at 37 °C for 20 min. The nucleus was counter-stained with 1 μg/mL DAPI and observed for fluorescence using an inverted fluorescent microscope (Olympus, Germany), and image alignment was done by Canvas imaging software.

**Photothermal transduction efficacy of MI PNPs:** Photothermal transduction efficacy was evaluated by irradiating 300 μL (0.4 mg/mL) of PMIR NPs with 808 nm laser (Shanghai Inter-Diff Optoelectronics Technology Ltd., Shanghai, China) and PC NPs, Milli-Q water, and PI NPs as controls. The temperature rise was recorded using a thermal probe at 0, 2, 4, 6, 8, and 10 min and temperature vs. time was plotted for P, PM, PIR NPs, and water.

**Biocompatibility of PNPs:** In vitro biological compatibility studies were carried out with HRMEV (human retinal micro vascular endothelial cells) at 1 × 10^4^ cells per well. After 24 h, P, PM, PIR, and PMIR NPs were diluted at 10–100 μg/mL in media. After incubation, cell viability was observed using an MTT assay. 

**Plasmid DNA damage assay:** The potential to cleave DNA was evaluated by using pSuper plasmid DNA as a substrate in Tris–HCl (50 mM, pH 7.4) in the presence of MI PNPs (1 µg/mL) with or without NIR irradiation [48]. Further, the process of DNA cleavage that was induced by MI PNPs and irradiation was analyzed using agarose gel electrophoresis and images were captured using a Gel Doc 2000 imager system (Bio-Rad, Munich, Germany).

**Protein analysis:** Y79 cell lysates after the PTT treatment were analyzed by 10% SDS polyacrylamide gel electrophoresis. After boiling for 5 min in equal amounts of sample buffer, each sample was electrophoresed by SDS-PAGE in a Bio-Rad tank at maximum voltage and a constant current of 25 mAmp. After 2 h, the gels were de-stained and filmed digitally. The Gel Documentation System (Gel-Doc-XR; Bio-Rad, Hercules, CA, USA) was used to determine the protein band intensity, then standardized and compared to the control group to obtain the final results. The Quantity One program from the Discovery Series was used to measure the bands on the gels (version 4.5.2, Bio-Rad, Hercules, CA, USA.).

**Western blotting:** Cell lysate proteins were dispersed and isolated through electrophoresis with 12% SDS-PAGE and transferred to a PVDF membrane. These were probed with fixed antibodies, and analyzed through chemiluminescence. The total protein amount was estimated through the BCA kit [49,50].

**Anti-fungal activity:** The PTT mediated antifungal effects of MI PNPs were estimated in *Candida albicans* with respect to controls (untreated). The fungal culture *Candida* inoculum prepared by Clinical Laboratory Standards Institute (CLSI) standardized methods to perform the experiment. The inoculum was treated with 5 µL MI PNPs and incubated for 4 h. Control groups were incubated without any further treatment for 24 h. The treated groups were then subjected to 808 nm laser irradiation for 5 min. The plates were further incubated at 37 °C for 48 h and captured under microscope.

**Statistical analysis:** All the statistical analysis were performed using -two-way ANOVA. (ns, *p* > 0.05, * *p* < 0.05, ** *p* < 0.01, *** *p* < 0.001, **** *p* < 0.0001). 

## 3. Results and Discussion

***Identification and Preparation of bioactive fraction from M. oleifera:***

The bioactive fraction of *M. oleifera* was identified by qualitative secondary metabolite detection tests. Preliminary phytochemical screening results of the various fractions of *M. oleifera* indicated that the dichloromethane fraction was rich in secondary metabolites such as flavonoids, alkaloids, terpenoids, and glycosides (Figure S1). These metabolites were reported to possess unique cytotoxic properties against various cancer models. The DCM fraction was also found to possess derivatives of chlorophyll as seen in the FTIR analysis of DFM wherein bonds at 1650 cm^−1^ could correspond to the C=O bond of the aldehyde group, 2910 cm^−1^ corresponds to C-H bond, and 3420 cm^−1^ corresponds to the OH group present in the fraction (Figure S2). The chlorophyll derivatives were reported to induce reactive oxygen species (ROS) in cancer cells and cause death and, therefore, extensively studied as photodynamic agents. The presence of cancer cell cytotoxic secondary metabolites and chlorophyll derivatives encouraged us to choose the DFM fraction for our further studies. The DFM fraction of *M. oleifera* was further referred to as the bioactive fraction of *M. oleifera* (BM).

***GCMS analysis of the bioactive fraction of M. oleifera:***

The bioactive fraction was subjected to GC-MS chromatography to understand the types of secondary metabolites that were present. The GC-MS chromatogram of BM indicated seven major compounds (Figure S3A,B). The matched molecules and their retention time (RT) and chemical structures were indicated (Figure S2C). Most of these compounds were well studied for their potent anti-cancer properties in various cancer models. It can be concluded that these compounds, along with the PTT, could be responsible for the synergistic observations in retinoblastoma cell lines.

***Synthesis and Characterization of the bioactive fraction of M. oleifera and M. oleifera/IR820 loaded polymeric nanoformulations:***

**Figure 1:**
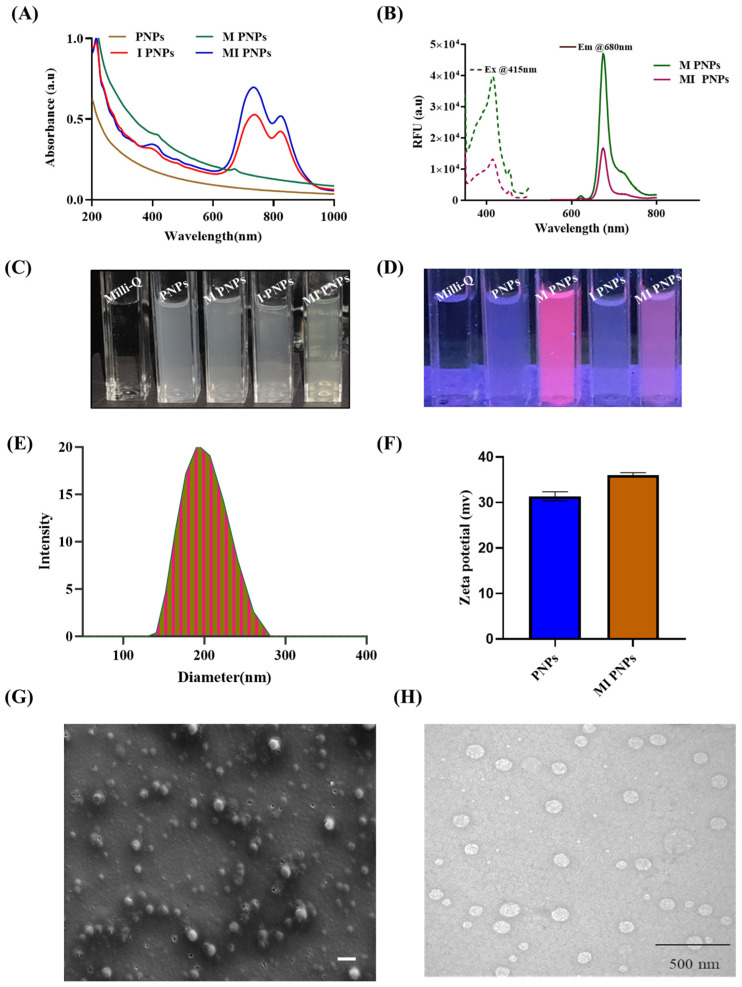
Characterization of the MI PNPs. (**A**) UV-Vis spectra of the blank, M, I, and MI PNPs. (**B**) Fluorescent spectra of the M and MI PNPs (ex/em: 415/680 nm). (**C**,**D**) Representative images of the various PNPs under bright and UV light. (**E**,**F**) DLS and zeta potential graphs for the MI PNPs. (**G**,**H**) SEM and TEM images of the MI PNPs.

Since the bioactive fraction of *M. oleifera* (BM) contains hydrophobic secondary metabolites and chlorophyll moieties, it is insoluble in water. Therefore, a polymeric nanoformulation of *M. oleifera (M PNPs)* was prepared and characterized. UV-visible absorption measurements were taken to explore the interaction of the M/IR 820 dye with the NPs. The absorption spectrum of M PNPs (Figure 1A) revealed peaks at 400 and 680 nm corresponding to chlorophyll A. I PNPs exhibited two peaks: one at 845 and the other at 750/ 765 nm. The UV spectra of MI PNPs showed equivalent peaks at 400, 750, and 845 nm indicating the successful incorporation of BM/IR820 into PNPs without harming any properties. M PNPS exhibited red fluorescence (SI Figure 1C,D) when excited at 400 nm (Figure 1B). The aqueous M PNPs formulation was uniform, and no precipitates were observed (Figure 1C,D). The average hydrodynamic diameter of the MI PNPs was found to be in the range of 180–220 nm (Figure 1E). The FE-SEM and TEM analysis indicated that the MI PNPs were spherical and 160–200 nm in size (Figure 1F,G). The zeta potential of MI PNPs was found to be cationic and, therefore, may aid in the elimination or damaging the negatively charged cancer cells through enhancing the cancer cellular internalization (Figure 1H).

***Photothermal efficacy studies of the MI PNPs:***

**Figure 2:**
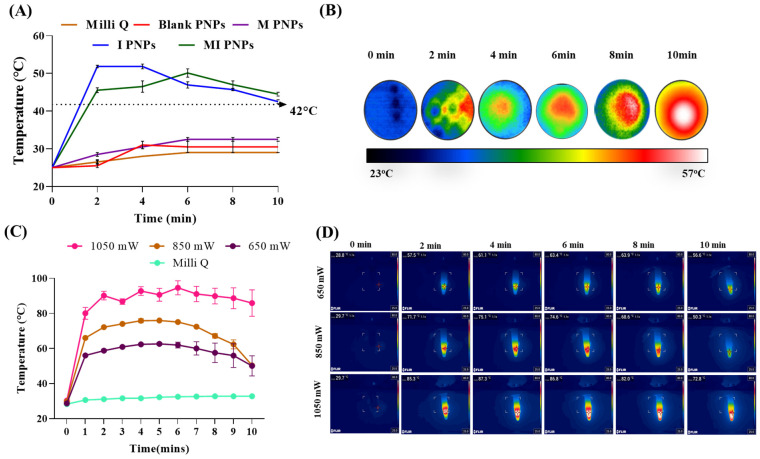
Photothermal transduction efficacy studies of the MI PNPs during exposure to NIR 808 nm light. (**A**) Line graph indicating the temperature profiles of the various PNPs and controls. (**B**) Representative thermal images indicating the temperature increments. (**C**) Line graph indicating the temperature profiles of the various PNPs and controls for different power intensities of the NIR light. (**D**) Representative thermal images indicating the temperature increments for different power intensities of the NIR light.

The photothermal efficacy studies of the MI PNPs were studied by exposing them to an NIR light exposure and subsequently recording the temperature increment at various defined time points. The Milli-Qq water, blank PNPs, M PNPs, and I PNPs groups were included as reference controls. The experimental recordings indicated that the PNPs containing IR-820 (I PNPs) and a combination of IR-820/M (MI PNPs) had exhibited a temperature of 53 °C and 51 °C, respectively, whereas the Milli-Q control, blank PNP, and M PNPs groups displayed a maximum temperature of 32 °C upon 5 min of NIR light exposure (Figure 2A). The corresponding thermal images also exhibited a similar pattern of thermal signals (Figure 2B). To further understand the effect of NIR light-mediated thermal response, we have exposed the MI PNPs at different power intensities ranging from 650 mW to 1050 mW. We observed that the MI PNPs group exposed to 1050 mW had exhibited the highest thermal response, followed by 850 and 650 mW exposed groups when observed from 0 to 10 min. Overall, the results indicated that the photothermal transduction efficiency of MI PNPs is proportional to the power intensity of the NIR light (Figure 2C,D). This property is useful in achieving the desired optimal photothermal therapeutic response.

***Photothermal degradation and release studies of MI PNPs:***

**Figure 3:**
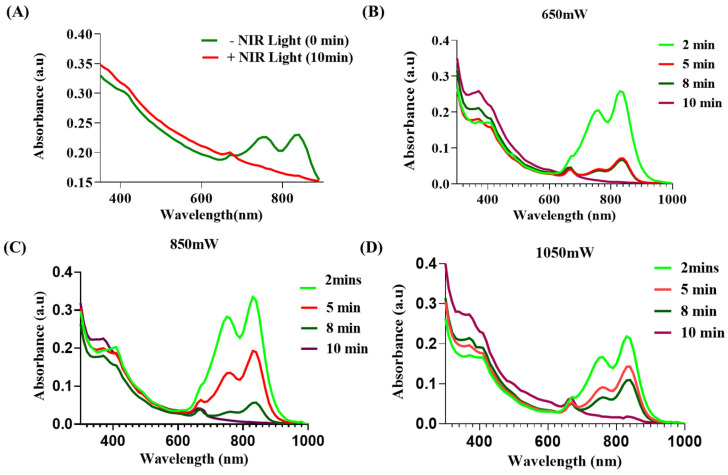
PTT-mediated release/degradation studies of the MI PNPs during exposure to NIR 808 nm light. (**A**) Line graph indicating the UV-vis spectra before and post-NIR light irradiation. (**B**–**D**) UV-vis spectra of MI PNPs indicating the release/degradation using various power intensities of NIR light.

To understand the NIR light-mediated degradation and release profile of MI PNPs, a UV-visible spectrophotometric technique was used. The MI PNPs were irradiated with 808 nm NIR laser for 10 min. The changes in the absorbance spectra were evaluated further. MI PNPs encompass both the bioactive fraction of *M. oleifera* (absorption at 400 and 680 nm) and IR820 dye (absorption at 800 nm). After exposure to NIR light, the intensity of the characteristic peak of IR820 dye has significantly reduced, indicating degradation. In contrast, the intensity of *M. oleifera* absorbance increased after 10 min of NIR exposure indicating its release from the polymeric nano-delivery system (Figure 3A). To further confirm the same, we have exposed the MI PNPs with various power intensities of NIR light for different intervals of time. The UV-visible spectra of the samples clearly indicated that the absorption intensity of the IR820 dye decreased, whereas the bioactive fraction of *M. oleifera* exhibited an increased absorption intensity as the time of exposure progressed (Figure 3B–D). From the results, it can be deciphered that the NIR light-mediated heat has contributed to the degradation/lysis of polymeric shells and facilitated the release of bioactive *M. oleifera* fraction.

***Cellular internalization ability of the M. oleifera/IR820 loaded polymeric nanoformulations:***

**Figure 4:**
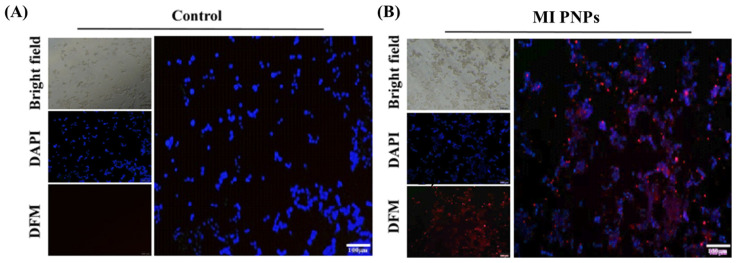
Fluorescent microscopy images of (**A**) untreated cells and (**B**) cells treated with MI PNPS indicating the cellular internalization efficacy of the MI PNPs.

The fluorescence property of chlorophyll derivatives that are present in the bioactive fraction of *M. oleifera* was utilized to understand the cellular internalization ability of the MI PNPs using the Y79 retinoblastoma cell lines. The fluorescent microscopy imaging indicated that the MI PNPs exhibited a red fluorescence within the cytoplasmic region, whereas the control group cells had not displayed a fluorescence signal in the cytoplasm. However, the nuclear staining using DAPI indicated a blue signal in both groups (Figure 4A,B). This shows the theranostic property of the developed nanoformulation, wherein the nanosystem is used both for imaging and therapeutics.

***Photoacoustic imaging efficacy of MI PNPs using phantom experiments:***

Non-invasive photoacoustic imaging (PAI) techniques can access molecular and cell architectures more precisely than other modalities. Recently PAI has gained prominence in the differential diagnosis of tumor cells and demonstrated practical capabilities in ophthalmology for intraocular tumor imaging at micron-level resolutions. Therefore, PAI-compatible materials can be crucial in the overall theranostics of retinoblastoma. Therefore, the PA imaging compatibility of the MI PNPs was tested using phantom experiments. The FDA-approved dye indocyanine green (ICG) and Milli-Q water samples were used as reference standard and control groups. 

**Figure 5:**
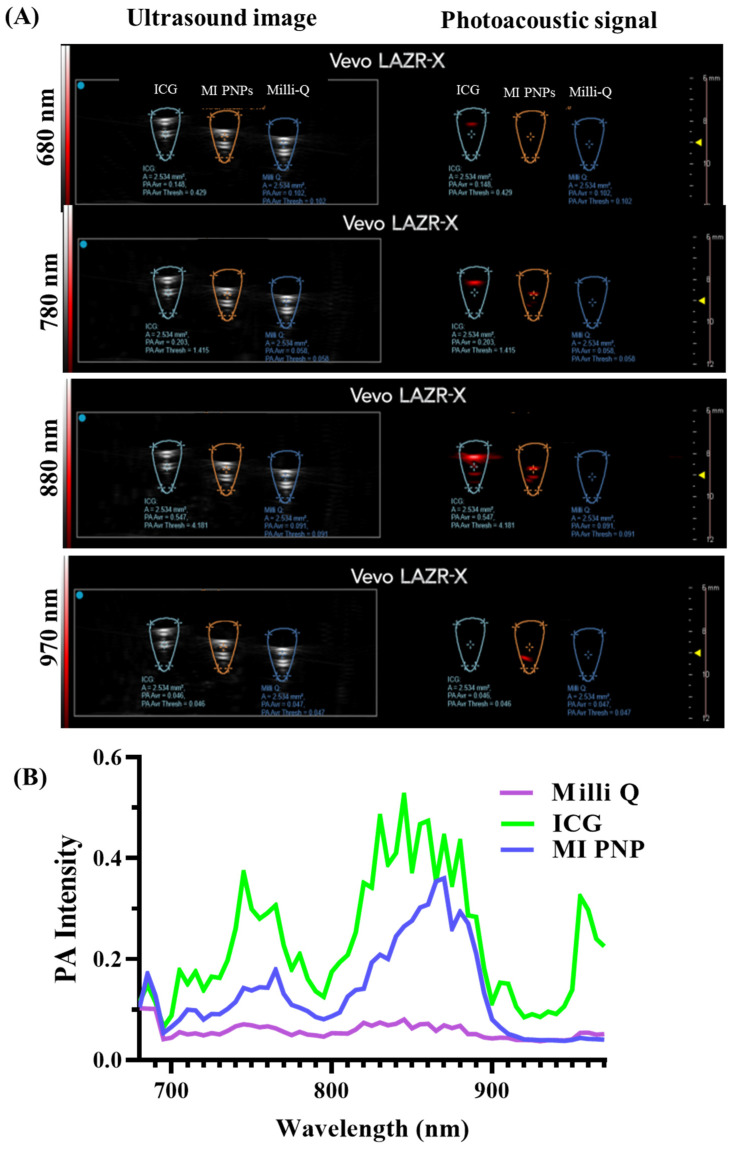
Phantom PA imaging of the MI PNPs compared to ICG and Milli-Q water. (**A**) The ultrasound/ PA signal images of the samples at different wavelengths. (**B**) The line graph spectra indicate the PA signal in the scan window.

The PA signal is shown in red, and the US signal from the tubes is shown in grey. The scan in the photoacoustic mode indicated that the standard ICG sample signal intensity gradually increased and was maximum at 880 nm wavelength and then eventually decreased. The MI PNPs sample also exhibited a similar and comparable signal to the standard ICG samples, whereas the control sample (Milli-Q water) signal was not detectable in the PA model (Figure 5A). The image analysis indicates that the MI PNPs were detectable in the phantom imaging tubes indicating their applicability as PA agents. The average photoacoustic intensity was calculated in the selected regions of interest (ROIs) and then plotted against the free IR780, maintain the concentrations constant. We observed a comparable PA intensity of MI PNPs with the ICG reference tube (Figure 5B). A stable increase in the intensity was observed till 815 nm, followed by a gradual reduction in the PA intensity. The phantom experiment revealed that the developed MI PNPs exhibits significant PA imaging potential, which uniquely combines the benefits of both ultrasound and NIR light, resulting in consistent absorption.

***Biocompatibility- and photothermal-mediated cytotoxicity studies of the MI PNPs:***

**Figure 6:**
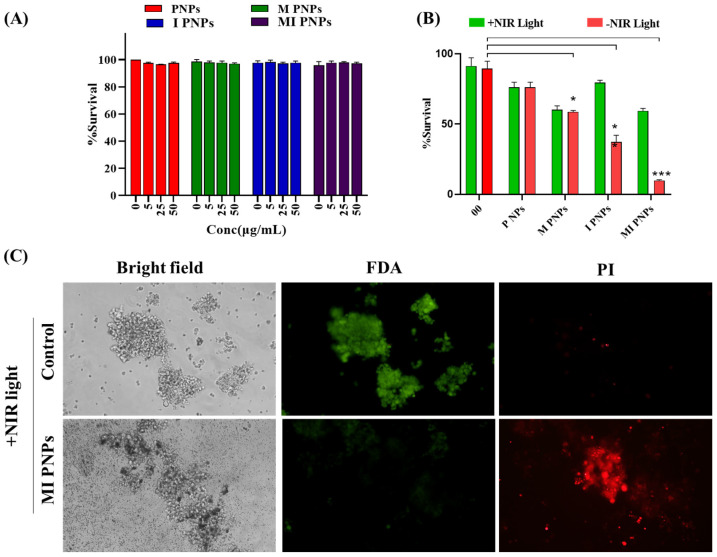
(**A**) Biocompatibility studies of the MI PNPs at various concentrations in HRMEC cell lines. (**B**) Photothermal-mediated cytotoxicity studies of the MI PNPs and reference controls in the presence or absence of the NIR light. (**C**) Fluorescent microscopy images of the FDA/PI-stained cells after treatment and exposure to NIR 808 nm light. (Scale bar represents 100 µm). A two-way ANOVA was used to perform the statistical analysis in (**B**). * *p* < 0.05 and *** *p* < 0.001.

The biocompatibility investigation of HRMEV cells revealed that PNPs showed no toxicity in the concentration range of 5 to 50 µg/mL, resulting in only 5% of cell death (Figure 6A).

Overall, the studies indicated that the MI PNPs were found to be biocompatible. Further PTT-mediated cytotoxicity studies were conducted in the Y79 retinoblastoma cell lines. The percentage of viable cells was maximum in the control groups or treatment groups without NIR laser irradiation. The groups that were exposed to laser irradiation achieved a variety of outcomes. M PNPs did not allow the drug to be accessed or released from the particles in the group that received this treatment. The laser treatment of I PNPs resulted in significant cell death, with cell death rates approaching 50%. The MI PNPs group that received NIR light experienced the highest amount of cell death. The cell fatality rate exceeded 80%, suggesting that IR PNPs had a significant inhibitory effect on tumor cell proliferation. The microscopic live/dead (FDA/PI) assay of MI PNPs also indicated a significantly more cytotoxic effect than the control group (Figure 6B). The green fluorescence (FDA) indicated the live cells were high in the control group and high in the case of the MI PNPs exposed to the NIR light group. Similarly, the opposite phenomenon was observed with the propidium iodide stain indicating a high number of dead cells in the MI PNPs group (Figure 6C). Overall, it can be concluded that the PTT combined with MI PNPs demonstrated synergistic benefits compared to solo treatments (M and I PNPs). The combination of IR-820 and BM through the polymeric nano-delivery system may have cellular internalization leading to optimized hyperthermia resulting in the synergistic cytotoxic effect of MI PNPs. MI PNPs were selective in blocking cancer cell proliferation over extended periods, as demonstrated by these data.

***Mechanism studies of the MI PNPs:***

Proteins are also sensitive to reactive oxygen species (ROS) oxidation. Several amino acids, including arginine, histidine, methionine, and cysteine, are susceptible to oxidation in an antioxidant insufficiency. Oxidative protein damage has been shown to have a crucial part in cytotoxicity.

**Figure 7:**
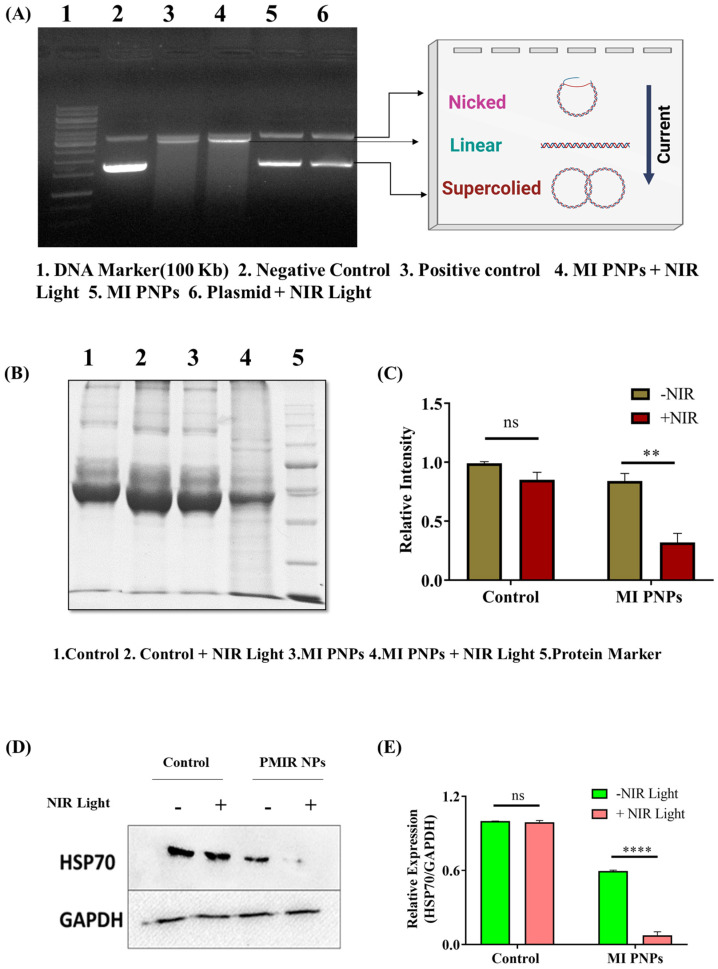
Protein/DNA analysis for the samples that were treated with the MI PNPs in the presence or absence of the NIR light. (**A**) Plasmid denaturation assay indicating the formation of nicked, linear, or supercoiled plasmid content. (**B**) Protein denaturation assay. (**C**) bar graph indicating the relative intensities. (**D**) Western blot analysis for the cell lysates that were treated with the MI PNPs in the presence or absence of the NIR 808 nm light. A two-way ANOVA was used to perform the statistical analysis in (**C**,**E**) where ** corresponds to *p* < 0.01 and **** corresponds to *p* < 0.0001.

As such, the measurement of protein oxidation has been a sensitive tool for evaluating oxidative protein damage in various situations. In this study, densitometric analysis of protein bands and quantified gel images revealed that Y79 cells that were treated with MI PNPs when exposed to NIR light had shown protein denaturation compared with controls (Figure 7B,C). This could be due to the heat that is generated by NIR dye. HSP70 is a member of the HSP family, involved in the folding and functioning of numerous proteins. It is also critical for tumor cell survival. The role of heat-shock proteins after PTT treatment was evaluated. We found that control groups had higher levels of HSP expression. According to these findings, high temperatures induced HSP expression in the body. The expression of HSP70 was suppressed due to the presence of DFM when the ambient temperature increased. Lowering the levels of HSP70 in MI PNPs with NIR irradiated cells (Figure 7D,E) aids the heat sensitivity of the tumor cells.

## 4. Conclusions

In this study, we report the role of a bioactive fraction from *M. oleifera* in the downregulation of HSP70 expression for a synergistic photothermal therapy in retinoblastoma cell lines. The hydrophobic bioactive fraction from *M. oleifera* was successfully incorporated into a polymeric nano-delivery system. It was tuned photo-thermally active by co-encapsulating IR820 dye, which also facilitated the PTT-mediated release of a potent anti-cancer fraction from the nanosystem. The in vitro cellular uptake efficiency of the bioactive fraction of *M. oleifera* was enhanced when loaded into a polymeric nano-delivery system, thereby improving the overall efficiency of cancer therapy. The incorporation of both the bioactive *M. oleifera*/IR820 together into polymeric nanosystems (i.e., MI PNPs) resulted in a significantly reduced cell viability of retinoblastoma cells when compared to individual PTT or bioactive fraction of *M. oleifera* treatment. The mechanism of this synergistic anti-cancer activity was elucidated to be downregulation of HSP70 expression facilitated by the bioactive fraction of *M. oleifera*. Although no purified active component of the plant was used, GC-MS analysis of the DFM revealed seven major secondary metabolites, based on the retention time, which have been well reported for potential anti-cancer activities. Also, in our initial trials, significant DNA damage was observed in the plasmid denaturation assay, indicating an apoptotic form of cell death. However, further in-depth studies such as flow cytometry, Western blot, or qRT-PCR for the evaluation of expression of apoptotic/autophagic markers are required to elucidate the role of DFM/IR820 mediating PTT in the synergistic cell death. Overall, the combination of a bioactive phytochemicals with photothermal therapy serves as a potential therapeutic modality to overcome the limitations of existing monotherapies.

## Data Availability

Not applicable.

## Supplementary Materials

The following supporting information can be downloaded at: https://www.mdpi.com/article/10.3390/pharmaceutics15020475/s1, Figure S1: Phytochemical screening of various extracts of M. oleifera; Figure S2: FTIR spectra of DFM; Figure S3: (A) A schematic depicting the extraction procedure of DFM, (B) GC-MS analysis of the DFM and (C) List of components present in the DFM and their medicinal uses; Figure S4: (A) UV-Vis absorbance, (B) Fluorescence spectrum, (C) Photographs of DFM under bright light and UV light & (D) Radical scavenging activity of DFM; Figure S5: Cell viability of (A) HRMEV and (B) Y79 cell lines treated with DFM; Figure S6: Hydrodynamic diameter and Zeta potential of the synthesized nanoparticles; Figure S7: Anti-fungal activity of the synthesized MI PNPs nanoparticles upon NIR laser irradiation.

## References

[1] Deo S.V.S., Sharma J., Kumar S. (2022). GLOBOCAN 2020 Report on Global Cancer Burden: Challenges and Opportunities for Surgical Oncologists. Ann. Surg. Oncol..

[2] Parulekar M.V. (2010). Retinoblastoma—Current treatment and future direction. Early Hum. Dev..

[3] Dimaras H., Kimani K., Dimba E.A.O., Gronsdahl P., White A., Chan H.S.L., Gallie B.L. (2012). Retinoblastoma. Lancet.

[4] Dimaras H., Corson T.W., Cobrinik D., White A., Zhao J., Munier F.L., Abramson D.H., Shields C.L., Chantada G.L., Njuguna F. (2015). Retinoblastoma. Nat. Rev. Dis. Prim..

[5] White L. (1991). Chemotherapy in retinoblastoma: Current status and future directions. Am. J. Pediatr. Hematol. Oncol..

[6] Kaewkhaw R., Rojanaporn D. (2020). Retinoblastoma: Etiology, Modeling, and Treatment. Cancers.

[7] Jabbour P., Chalouhi N., Tjoumakaris S., Gonzalez L.F., Dumont A.S., Chitale R., Rosenwasser R., Bianciotto C.G., Shields C. (2012). Pearls and pitfalls of intraarterial chemotherapy for retinoblastoma: A review. J. Neurosurg. Pediatr..

[8] Zanaty M., Barros G., Chalouhi N., Starke R.M., Manasseh P., Tjoumakaris S.I., Shields C.L., Hasan D., Bulsara K., Rosenwasser R.H. (2014). Update on Intra-Arterial Chemotherapy for Retinoblastoma. Sci. World J..

[9] Stacey A.W., De Francesco S., Borri M., Hadjistilianou T. (2021). The Addition of Topotecan to Melphalan in the Treatment of Retinoblastoma with Intra-arterial Chemotherapy. Ophthalmol. Retin..

[10] Kiratli H., Koç I., Öztürk E., Varan A., Akyüz C. (2020). Comparison of intravitreal melphalan with and without topotecan in the management of vitreous disease in retinoblastoma. Jpn. J. Ophthalmol..

[11] Delrish E., Jabbarvand M., Ghassemi F., Amoli F.A., Atyabi F., Lashay A., Soleimani M., Aghajanpour L., Dinarvand R. (2021). Efficacy of topotecan nanoparticles for intravitreal chemotherapy of retinoblastoma. Exp. Eye Res..

[12] Nemeth K.M., Federico S., Carcaboso A.M., Shen Y., Schaiquevich P., Zhang J., Egorin M., Stewart C., Dyer M.A. (2011). Subconjunctival carboplatin and systemic topotecan treatment in preclinical models of retinoblastoma. Cancer.

[13] Cieślik K., Rogowska A., Hautz W. (2022). Focal therapies for retinoblastoma. Klin. Ocz. Acta Ophthalmol. Pol..

[14] Shields C.L., Honavar S.G., Meadows A.T., Shields J.A., Demirci H., Singh A., Friedman D.L., Naduvilath T.J. (2002). Chemoreduction plus focal therapy for retinoblastoma: Factors predictive of need for treatment with external beam radiotherapy or enucleation11InternetAdvance publication at ajo.com April 8, 2002. Am. J. Ophthalmol..

[15] Mudigunda S.V., Pemmaraju D.B., Paradkar S., Puppala E.R., Gawali B., Upadhyayula S.M., Vegi Gangamodi N., Rengan A.K. (2022). Multifunctional Polymeric Nanoparticles for Chemo/Phototheranostics of Retinoblastoma. ACS Biomater. Sci. Eng..

[16] Yang X.-J., Li X.-L., Chen H.-Y., Xu J.-J. (2019). NIR-Activated Spatiotemporally Controllable Nanoagent for Achieving Synergistic Gene-Chemo-Photothermal Therapy in Tumor Ablation. ACS Appl. Bio Mater..

[17] Padalkar M.V., Pleshko N. (2015). Wavelength-dependent penetration depth of near infrared radiation into cartilage. Analyst.

[18] Rengan A.K., Bukhari A.B., Pradhan A., Malhotra R., Banerjee R., Srivastava R., De A. (2015). In vivo analysis of biodegradable liposome gold nanoparticles as efficient agents for photothermal therapy of cancer. Nano Lett..

[19] Appidi T., PS R., Chinchulkar S.A., Pradhan A., Begum H., Shetty V., Srivastava R., Ganesan P., Rengan A.K. (2022). A plasmon-enhanced fluorescent gold coated novel lipo-polymeric hybrid nanosystem: Synthesis, characterization and application for imaging and photothermal therapy of breast cancer. Nanoscale.

[20] Jogdand A., Alvi S.B., Rajalakshmi P.S., Rengan A.K. (2020). NIR-dye based mucoadhesive nanosystem for photothermal therapy in breast cancer cells. J. Photochem. Photobiol. B Biol..

[21] Farhat W., Yeung V., Ross A., Kahale F., Boychev N., Kuang L., Chen L., Ciolino J.B. (2022). Advances in biomaterials for the treatment of retinoblastoma. Biomater. Sci..

[22] Bin L., Du Y., Zhang Y., Xiao Q., Chen X., Liu Z., Du Z. (2022). Phase-changeable nanoparticles loaded with FeⅢ-tannic acid/paclitaxel for retinoblastoma treatment. J. Drug Deliv. Sci. Technol..

[23] Zheng W., Li X., Zou H., Xu Y., Li P., Zhou X., Wu M. (2022). Dual-Target Multifunctional Superparamagnetic Cationic Nanoliposomes for Multimodal Imaging-Guided Synergistic Photothermal/Photodynamic Therapy of Retinoblastoma. Int. J. Nanomed..

[24] Li M., Bian X., Chen X., Fan N., Zou H., Bao Y., Zhou Y. (2022). Multifunctional liposome for photoacoustic/ultrasound imaging-guided chemo/photothermal retinoblastoma therapy. Drug Deliv..

[25] Mendes R., Pedrosa P., Lima J.C., Fernandes A.R., Baptista P.V. (2017). Photothermal enhancement of chemotherapy in breast cancer by visible irradiation of Gold Nanoparticles. Sci. Rep..

[26] Khademi R., Razminia A. (2020). Selective nano-thermal therapy of human retinoblastoma in retinal laser surgery. Nanomed. Nanotechnol. Biol. Med..

[27] Liu Y., Han Y., Chen S., Liu J., Wang D., Huang Y. (2022). Liposome-based multifunctional nanoplatform as effective therapeutics for the treatment of retinoblastoma. Acta Pharm. Sin. B.

[28] Russo E., Spallarossa A., Tasso B., Villa C., Brullo C. (2022). Nanotechnology for Pediatric Retinoblastoma Therapy. Pharmaceuticals.

[29] Evans C.G., Chang L., Gestwicki J.E. (2010). Heat shock protein 70 (hsp70) as an emerging drug target. J. Med. Chem..

[30] Sun T., Chen X., Wang X., Liu S., Liu J., Xie Z. (2019). Enhanced efficacy of photothermal therapy by combining a semiconducting polymer with an inhibitor of a heat shock protein. Mater. Chem. Front..

[31] Zhang G., Cheng W., Du L., Xu C., Li J. (2021). Synergy of hypoxia relief and heat shock protein inhibition for phototherapy enhancement. J. Nanobiotechnol..

[32] Cao Y., Ren Q., Hao R., Sun Z. (2022). Innovative strategies to boost photothermal therapy at mild temperature mediated by functional nanomaterials. Mater. Des..

[33] Fraiser L.H., Kanekal S., Kehrer J.P. (1991). Cyclophosphamide Toxicity. Drugs.

[34] Latchman J., Guastella A., Tofthagen C. (2014). 5-Fluorouracil toxicity and dihydropyrimidine dehydrogenase enzyme: Implications for practice. Clin. J. Oncol. Nurs..

[35] Chatterjee K., Zhang J., Honbo N., Karliner J.S. (2010). Doxorubicin cardiomyopathy. Cardiology.

[36] Dhamija E., Meena P., Ramalingam V., Sahoo R., Rastogi S., Thulkar S. (2020). Chemotherapy-induced pulmonary complications in cancer: Significance of clinicoradiological correlation. Indian J. Radiol. Imaging.

[37] Desai A., Qazi G., Ganju R., El-Tamer M., Singh J., Saxena A., Bedi Y., Taneja S., Bhat H. (2008). Medicinal Plants and Cancer Chemoprevention. Curr. Drug Metab..

[38] Shukla S., Mehta A. (2015). Anticancer potential of medicinal plants and their phytochemicals: A review. Braz. J. Bot..

[39] Greenwell M., Rahman P.K.S.M. (2015). Medicinal Plants: Their Use in Anticancer Treatment. Int. J. Pharm. Sci. Res..

[40] Tekayev M., Bostancieri N., Saadat K.A.S.M., Turker M., Yuncu M., Ulusal H., Cicek H., Arman K. (2019). Effects of Moringa oleifera Lam Extract (MOLE) in the heat shock protein 70 expression and germ cell apoptosis on experimentally induced cryptorchid testes of rats. Gene.

[41] Singh B.N., Singh B.R., Singh R.L., Prakash D., Dhakarey R., Upadhyay G., Singh H.B. (2009). Oxidative DNA damage protective activity, antioxidant and anti-quorum sensing potentials of Moringa oleifera. Food Chem. Toxicol..

[42] Tiloke C., Phulukdaree A., Anand K., Gengan R.M., Chuturgoon A.A. (2016). *Moringa oleifera* Gold Nanoparticles Modulate Oncogenes, Tumor Suppressor Genes, and Caspase-9 Splice Variants in A549 Cells. J. Cell. Biochem..

[43] Barhoi D., Upadhaya P., Barbhuiya S.N., Giri A., Giri S. (2021). Aqueous Extract of Moringa oleifera Exhibit Potential Anticancer Activity and can be Used as a Possible Cancer Therapeutic Agent: A Study Involving In Vitro and In Vivo Approach. J. Am. Coll. Nutr..

[44] Khan F., Pandey P., Ahmad V., Upadhyay T.K. (2020). *Moringa oleifera* methanolic leaves extract induces apoptosis and G0/G1 cell cycle arrest via downregulation of Hedgehog Signaling Pathway in human prostate PC-3 cancer cells. J. Food Biochem..

[45] Pemmaraju D., Appidi T., Minhas G., Singh S.P., Khan N., Pal M., Srivastava R., Rengan A.K. (2018). Chlorophyll rich biomolecular fraction of *A. cadamba* loaded into polymeric nanosystem coupled with Photothermal Therapy: A synergistic approach for cancer theranostics. Int. J. Biol. Macromol..

[46] Sanna D., Delogu G., Mulas M., Schirra M., Fadda A. (2012). Determination of Free Radical Scavenging Activity of Plant Extracts Through DPPH Assay: An EPR and UV—Vis Study. Food Anal. Methods.

[47] Pagano C., Perioli L., Baiocchi C., Bartoccini A., Beccari T., Blasi F., Calarco P., Ceccarini M.R., Cossignani L., di Michele A. (2020). Preparation and characterization of polymeric microparticles loaded with Moringa oleifera leaf extract for exuding wound treatment. Int. J. Pharm..

[48] Iliakis G., Rosidi B., Wang M., Wang H. (2006). Plasmid-based assays for DNA end-joining in vitro. Methods Mol. Biol..

[49] Kazeem G.O., Adedayo F.E., Thomas A.O. (2017). Hyperthermal-induced stress effects on survival and expression of heat shock protein (HSP) genes in Nile tilapia, Oreochromis niloticus fingerlings fed aqueous extract from Moringa oleifera leaf. Livest. Res. Rural Dev..

[50] Alvi S.B., Appidi T., Deepak B.P., Rajalakshmi P.S., Minhas G., Singh S.P., Begum A., Bantal V., Srivastava R., Khan N. (2019). The “nano to micro” transition of hydrophobic curcumin crystals leading to in situ adjuvant depots for Au-liposome nanoparticle mediated enhanced photothermal therapy. Biomater. Sci..

