# Supplementary material for: Bioactive Polymeric Nanoparticles of Moringa oleifera Induced Phyto-Photothermal Sensitization for the Enhanced Therapy of Retinoblastoma
Source: Pharmaceutics. 2023 Jan 31;15(2):475. doi: 10.3390/pharmaceutics15020475 (PMC9965703; doi:10.3390/pharmaceutics15020475)
Supplement: Supplementary file 1 [file pharmaceutics-15-00475-s001.zip › pharmaceutics-2121489-supplementary.pdf]

Bioactive polymeric nanoparticles of *Moringa oleifera* induced phyto-photothermal sensitization for the enhanced therapy of retinoblastoma

Sushma V Mudigunda[1#], Deepak B. Pemmaraju[2#], Sri Amruthaa Sankaranarayanan[1], Aravind Kumar Rengan[1*]

[1] Department of Biomedical Sciences, Indian Institute of Technology Hyderabad, Kandi, Telangana, India.
[2] Department of Pharmacology & Toxicology, National institute of pharmaceutical education & research (NIPER) Guwahati, Assam, India.
* Correspondence: aravind@bme.iith.ac.in; Tel.: (+91-40) 2301 – 6106).
# Contributed equally.

SUPPORTING INFORMATION

Phytochemicals	HFM	DFM	CFM	AFM
Flavonoids	-	+	-	+
Alkaloids	-	+	+	-
Terpenoids	+	+	-	-
Tannins	-	-	-	+
Glycosides	+	+	-	-
Saponins	-	-	+	-
Phenols	-	-	-	-
Steroids	-	-	-	-

Figure S1. Phytochemical screening of various extracts of *M. oleifera.*

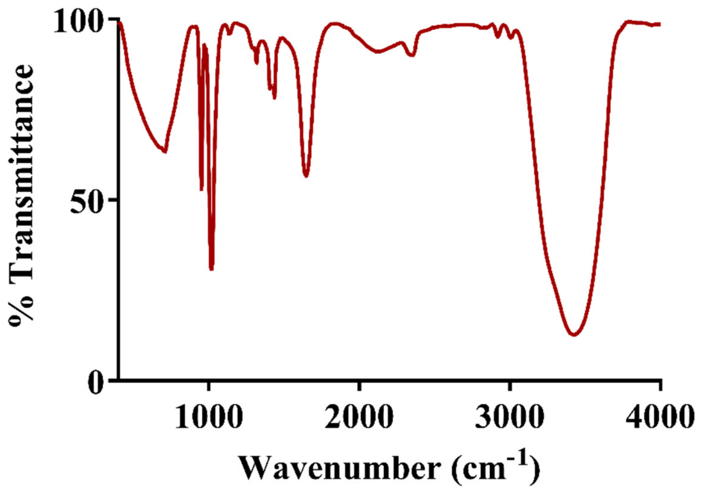

Figure S2. FTIR spectra of DFM.

A)

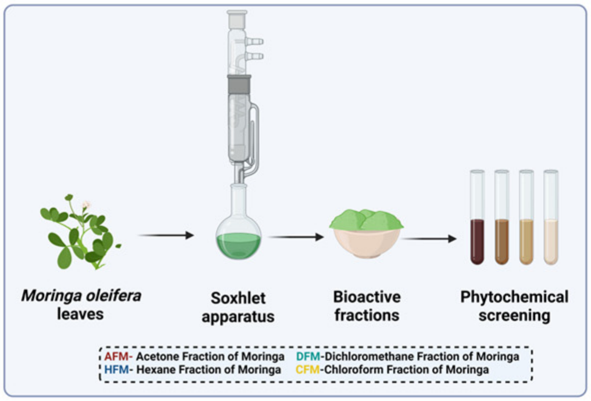

B)

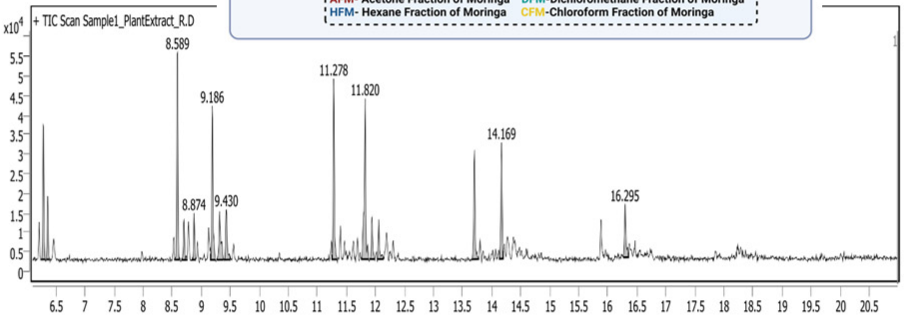

C)

S. No	Name	Structure	Reported Properties (Reference)
1	**Thiophene**	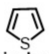 Thiophene	Chemotherapeutic agent,anti inflamatory,antimicrobial,antioxidant (67,68)
2	**dl-Stachydrine**	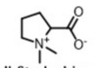 dl-Stachydrine	Treatment of fibrosis, cancers, uterine diseases, cardiovascular diseases, and inflammation (69)
3	**Tetrapentacontane**	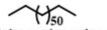 tetrapentacontane	Antioxidant and antimicrobial activity.(71)
4	**Solasonine**	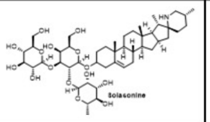	Anticancer agent inhibits cell proliferation, topical actinic keratosis treatment (73)
5	**Ginsenol**	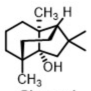 Ginsenol	Antiviral (anti-influenza virus activity), antibacterial, anti-inflammatory, and antifungal activities (74).
6	**Stannane**	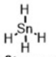 Stannane	Antifungal, antibacterial, and anticancer (76)
7	**Piperazine**	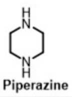 Piperazine	Derivatives of piperazine are used as antipsychotic, antidepressant, anxiolytic (72).

Figure S3. (A) A schematic depicting the extraction procedure of DFM, (B) GC-MS analysis of the DFM and (C) List of components present in the DFM and their medicinal uses.

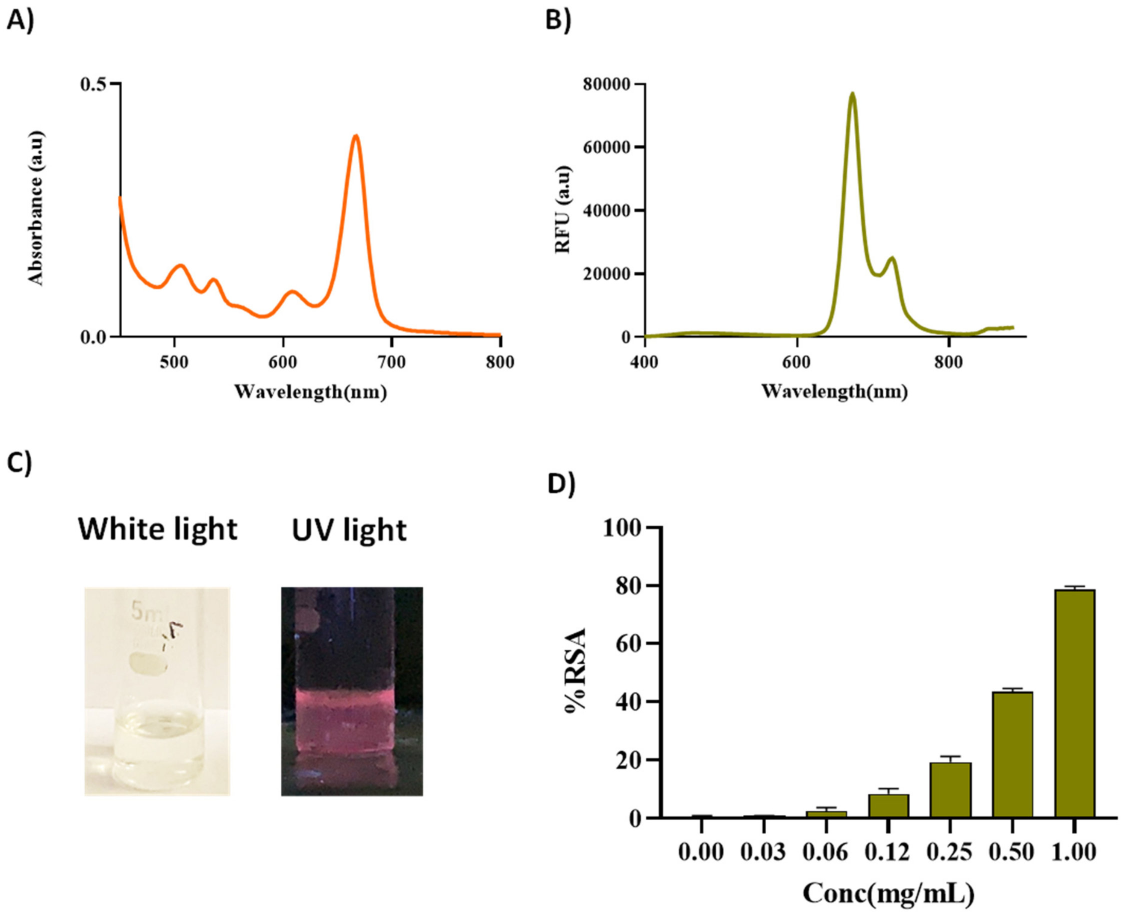

Figure S4. (A) UV- Vis absorbance, (B) Fluorescence spectrum, (C) Photographs of DFM under bright light and UV light & (D) Radical scavenging activity of DFM.

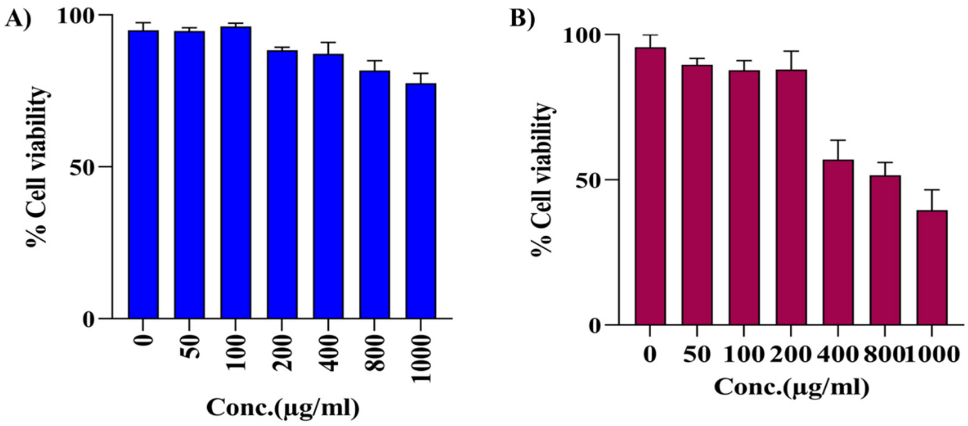

Figure S5. % Cell viability of (A) HRMEV and (B) Y79 cell lines treated with DFM.

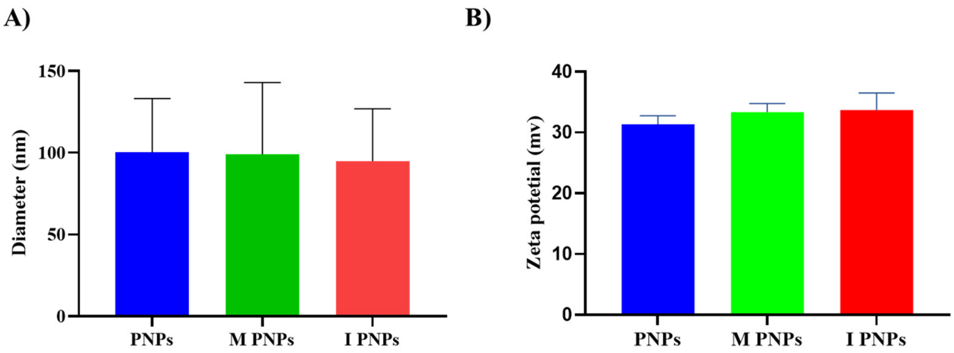

Figure S6. Hydrodynamic diameter and Zeta potential of the synthesized nanoparticles.

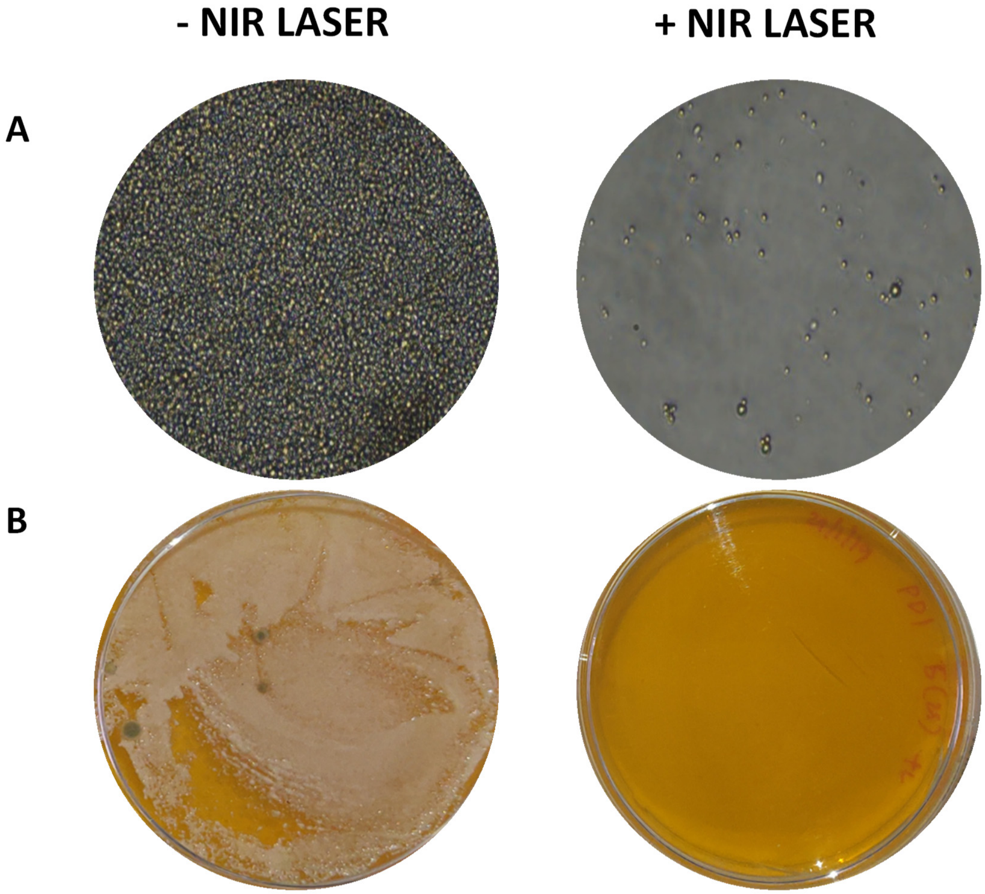

Figure S7. Anti-fungal activity of the synthesized MI PNPs nanoparticles upon NIR laser irradiation.
